# Disruption of protein geranylgeranylation in the cerebellum causes cerebellar hypoplasia and ataxia via blocking granule cell progenitor proliferation

**DOI:** 10.1186/s13041-023-01010-4

**Published:** 2023-02-13

**Authors:** Qi Cheng, Jing Wu, Yingqian Xia, Qing Cheng, Yinjuan Zhao, Peixiang Zhu, Wangling Zhang, Shihu Zhang, Lei Zhang, Yushan Yuan, Chaojun Li, Guiquan Chen, Bin Xue

**Affiliations:** 1 Medical School of Nanjing University, Jiangsu Key Laboratory of Molecular Medicine, Nanjing University, Nanjing, 210093 China; 2 Core Laboratory, Sir Run Run Hospital, Nanjing Medical University, Nanjing, 211166 China; 3 Department of Obstetrics, Obstetrics and Gynecology Hospital Affiliated to Nanjing Medical University, Nanjing, 210004 Jiangsu China; 4 Collaborative Innovation Center of Sustainable Forestry in Southern China, College of Forestry, Nanjing Forestry University, Nanjing, 210037 Jiangsu China; 5 Department of General Surgery, Affiliated Hospital of Nanjing University of Chinese Medicine, Nanjing, 210029 China; 6 Medical Imaging Center of Fuyang People’s Hospital, Fuyang, Anhui Province China; 7 State Key Laboratory of Reproductive Medicine and China International Joint Research Center On Environment and Human Health, Center for Global Health, School of Public Health, Nanjing Medical University, Nanjing, 211166 China; 8 Co-Innovation Center of Neuroregeneration, Nantong University, Nantong, 226001 China; 9 Collaborative Innovation Center for Cancer Personalized Medicine, Nanjing Medical University, Nanjing, 211166 China

**Keywords:** Ataxia, Cerebellum, Ggpps, Proliferation, Protein prenylation

## Abstract

The prenylation of proteins is involved in a variety of biological functions. However, it remains unknown whether it plays an important role in the morphogenesis of the cerebellum. To address this question, we generated a mouse model, in which the geranylgeranyl pyrophosphate synthase (*Ggps1*) gene is inactivated in neural progenitor cells in the developing cerebellum. We report that conditional knockout (cKO) of *Ggps1* leads to severe ataxia and deficient locomotion. To identify the underlying mechanisms, we completed a series of cellular and molecular experiments. First, our morphological analysis revealed significantly decreased population of granule cell progenitors (GCPs) and impaired proliferation of GCPs in the developing cerebellum of *Ggps1* cKO mice. Second, our molecular analysis showed increased expression of p21, an important cell cycle regulator in *Ggps1* cKO mice. Together, this study highlights a critical role of Ggpps-dependent protein prenylation in the proliferation of cerebellar GCPs during cerebellar development.

**Supplementary Information:**

The online version contains supplementary material available at 10.1186/s13041-023-01010-4.

## Introduction

The cerebellum is a brain region important for motor and non-motor functions [1–5]. It has been shown that abnormalities in the cerebellum are associated with several diseases such as cerebellar hypoplasia, ataxia, autism and Parkinson’s disease (PD) [6–8]. The cerebellum originates from the anterior hindbrain [9]. It is well known that the cerebellum of adult mice consists of 10 folia, and that each folium is composed of three layers, including a molecular layer (ML), a Purkinje cell (PC) layer (PCL) and a granular layer (GL) [8, 10, 11]. During early stages of the development, granule cell progenitors (GCPs) in the rhombic lip migrate to the external granular layer (EGL) and proliferate there so that the population gets expanded. GCPs then migrate to the internal granular layer (IGL) and differentiate into granule cells (GCs) [8]. Multiple molecular pathways have been reported to be involved in cerebellar development [12, 13].

Prenylation is an important protein modification way at the post-translational level in the cell [14, 15]. There are mainly two forms of protein prenylation, geranylgeranylation and farnesylation, in which a geranylgeranyl pyrophosphate (GGPP) or a farnesyl pyrophosphate (FPP) is attached to a cysteine at the C-terminus of a protein by a geranylgeranyl transferase (GGT) or a farnesyl transferase (FT), respectively [16–18]. GGPP is generated from FPP through a chemical reaction catalyzed by geranylgeranyl pyrophosphate synthase (Ggpps). Overall, Ggpps is a key regulator for protein prenylation in the cell. Abundant evidence has shown that the binding of cytoplasmic proteins to the plasma membrane is crucial for the activation of signaling pathways, and that the prenylation of a protein may facilitate the anchoring of it into the membrane and promote its interaction with other proteins [14, 18]. Furthermore, protein prenylation has been shown to be involved in a variety of cellular functions, including cell survival, cell proliferation, cell differentiation and cell migration [19–21].

Whereas Alzheimer’s disease (AD), PD, multiple sclerosis (MS) and amyotrophic lateral sclerosis (ALS) are well-known neurological disorders [22–25], abnormal protein prenylation has been widely observed in patients with these diseases [26–30]. Recent evidence has shown that protein prenylation may be important for synaptic plasticity in the hippocampus and dendritic morphogenesis in the cortex [31, 32]. A previous study demonstrated that geranylgeranyltransferase I (GGT) is essential for the dendritic development of PCs [33]. However, it remains largely unknown whether protein prenylation plays an important role in the morphogenesis of the cerebellum.

To address the above question, we took advantage of the human *Gfap-Cre* mouse [34] to generate a mouse model, in which *Ggps1* is inactivated in neural progenitor cells (NPCs) in the developing cerebellum. *Ggps1* conditional knockout (cKO) mice exhibited cerebellar hypoplasia and impairments in locomotion. *Ggps1* cKO mice displayed significantly decreased population of GCPs and reduced proliferation of GCPs in the developing cerebellum of *Ggps1* cKO mice. *Ggps1* cKO mice showed increased levels of cytosolic RhoA and p21, a cyclin-dependent kinase inhibitor, in the developing cerebellum. Together, these novel findings suggest that Ggpps may be crucial for the proliferation of cerebellar GCPs, the morphogenesis of the cerebellum and motor functions in mice.

## Materials and methods

### Animals

Male and female mice with the C57BL/6 J background were used in this study. They were purchased from the GemPharmatech (China). The *hGfap-Cre* transgenic mouse [34, 35] and the *Rosa26*^*mTmG/*+^ mouse [35, 36] were purchased from the Jackson Lab. The mice had ad libitum access to food and water, and they were group-housed in accordance with the regulations on mouse welfare and ethics of Nanjing University. The animal facility was maintained under a condition with a 12-h light/dark cycle. The day of vaginal plug detection in pregnant mice and the birth date of pups were defined as embryonic day 0.5 (E0.5) and postnatal day 0 (P0), respectively. The mice were sacrificed with CO_2_. All the mouse experiments were performed in accordance with the Guide for the Animal Care and Use Committee of the Model Animal Research Center of Nanjing University.

### Generation of *hGfap-Cre;Ggps1*^*fl/fl*^ mice

*Ggps1*^*fl/fl*^ mice were reported by us recently [37–40]. The *hGfap-Cre* mouse was backcrossed to C57BL/6 wildtype (WT) for at least six generations. To generate *Ggps1* cKO mice, *Ggps1*^*fl/fl*^ were bred with *hGfap-Cre* to obtain *hGfap-Cre;Ggps1*^*fl/*+^. The latter were then crossed to *Ggps1*^*fl/fl*^ to generate *hGfap-Cre;Ggps1*^*fl/fl*^ (*Ggps1* cKO) and *Ggps1*^*fl/fl*^ (control) mice.

### Genotyping

Crude genomic DNA was extracted from mouse tails using NaOH (50 mM) at 99 °C for 30 min. 1 μl of DNA extracts was used as a template and mixed with specific primers as well as Taq Master Mix (P112-01, Vazyme Biotech., China) for PCR amplification. PCR products were visualized by electrophoresis in 1.2% agarose gels with GelRed (TSJ003, Tsingke Biotech, China) to distinguish the *Ggps1*^*fl*^ allele and the WT allele. The primer information was as follows. For *Ggps1*^*fl*^, the forward primer was AATTGTGTGTGGTAGGGGTA and the reverse one was AACTTGCTTCA GAACTGAGC. For *Cre*, the forward primer was TGCCACGACCAAGTGACAG CAATG and the reverse one was AGAGACGGAAATCCATCGCTCG.

### Magnetic resonance imaging (MRI) analysis

Brain MRI was performed using mice at P16. The mice were anesthetized with 10% chloralic hydras (Sigma) by intraperitoneal injection. The head of a mouse was fixed and scanned with a 7 T Pharmascan system (Bruker, Germany). T2-weighted images were transversely acquired (field of view = 2 cm × 2 cm; slice thickness = 1 mm; repetition time = 2500 ms; echo time = 36 ms; flip angle = 129°; matrix = 256 × 256 pixels). MRI images were processed by ImageJ to measure the area of the cerebellum. Three MRI images were selected from each mouse for the area measurement. Five mice were used in each group.

### Sample collection

Brains from embryos and postnatal mice at different ages were dissected and then fixed in 4% paraformaldehyde overnight. The brains were dehydrated with graded ethanol (50%, 70%, 80%, 90%, 95% and 100%), infiltrated with xylene and embedded with paraffin. The paraffin blocks were sectioned sagittally (5 μm in thickness) using a microtome and mounted onto a glass slide. Sections were dried overnight.

### Hematoxylin and eosin (HE) staining

For HE staining, paraffin sections were deparaffinized with xylene, rehydrated with graded ethanol (100%, 100%, 90%, 80%, 70% and 50%) and then stained with hematoxylin and eosin. The sections were dehydrated with graded ethanol (50%, 70%, 80%, 90%, 95% and 100%) and xylene. They were sealed with a resin. Images for HE staining were captured using an Olympus microscope. Three images were selected for each mouse. At least three mice were used in each group.

### Immunohistochemistry (IHC)

A method described previously was used for IHC [36, 41, 42]. Brain sections were deparaffinized with xylene, rehydrated with graded ethanol and then boiled with a sodium citrate buffer (0.01 M) for antigen retrieval. The sections were permeabilized with 0.5% Triton X-100 for 15 min. The sections were blocked with 5% BSA at room temperature for 1 h, and then incubated with a primary antibody (NeuN, Millipore, MAB377; Calbindin, CST, 13176; Pax6, Biolegend, 901301; Ki67, Servicebio, GB121141; BrdU, Abcam, ab6326) at 4 °C overnight. Subsequently, the sections were washed for three times with phosphate buffered saline with 0.1% Tween-20 (PBST) and incubated with a fluorescence secondary antibody for 1 h at room temperature. The sections were stained with DAPI (1 μg/ml) for 5 min to label the nucleus. A drop of a 50% glycerol solution was placed on the slide, which was then covered with a coverslip. Fluorescence images were captured using an Olympus FV3000 confocal laser scanning microscope. Image ProPlus was used to count positive cells in a counting unit (200 μm × 200 μm for NeuN + cells in the IGL), in a rostral cerebellar lobule (for Pax6-positive (Pax6 +) cells, Pax6 + /BrdU + cells, Pax6 + /Ki67 + cells in the EGL, and Blbp + cells in the whole lobule) or in the cerebellum (for Calbindin + cells). The cell number for each section was then averaged across 3 sections to make the mean value. At least three mice were analyzed per group.

### BrdU labeling

We conducted this experiment using a method described recently [43, 44]. Briefly, BrdU (B5002, Sigma-Aldrich) was intraperitoneally injected to dams at E17.5 or pups at P0 with the concentration of 100 mg/kg. Brains were collected 30 min after the injection for E17.5 or 2 h after the injection for P0.

### Immunoblotting

The cerebellum was dissected from each mouse. Tissues were homogenized in cold radio-immunoprecipitation assay (RIPA) lysis buffer containing protease and phosphatase inhibitors (Thermo). Lysates were cleared by centrifugation (12,000 rpm for 20 min). The concentration of protein samples was determined using a standard BCA Protein Assay (Thermo). Loading protein samples were mixed with a loading buffer and boiled at 99 °C for 5 min. 30 μg total protein samples were resolved in a 10% or 12% SDS–PAGE for electrophoresis. Proteins in an electrophoresis gel were transferred to polyvinylidene fluoride membranes (Roche). The membrane was blocked with 5% non-fat milk (w/v) for 1 h and incubated with one of the following primary antibodies overnight at 4 °C: Ggpps (Santa Cruz, sc-271680), Pax6 (Biolegend, 901301), Rap1A (Santa Cruz, sc-65), Rap1 (Santa Cruz, sc-398755), RhoA (Santa Cruz, sc-418), p21 (CST, 64016S), Atp1a1 (Proteintech, 55187-1-AP), β-actin (ABclonal, AC026). After it was washed out for three times, the membrane was incubated with a horseradish peroxidase (HRP)-conjugated secondary antibody for 1 h at room temperature. The membrane was visualized using an enhanced chemiluminescence system (Tanon-4600, Tanon, China). ImageJ was used to quantify intensities for targeted protein bands.

### Cell fractionation

Cell fractionation was performed using a Membrane and Cytosol Protein Extraction Kit (P0033, Beyotime, China). Briefly, tissues were homogenized in cold reagent A containing PMSF, followed by centrifugation (700×*g*, 10 min, 4 °C) to remove nuclear components and unbroken cells. The supernatants were fractionated with a centrifugation at 14,000×*g* for 30 min at 4 °C. After cytosolic fractions were obtained from the supernatants, reagent B was added to collect membrane fractions. Equal volumes of cytosol and membrane fractions were used to conduct Western blotting.

### Quantitative real-time PCR (qRT-PCR)

Total RNAs were isolated from the cerebellum of a mouse using a TRIzol reagent (9109, Takara, China). The purity and concentration of RNAs were determined using a spectrophotometer (NanoDrop, ND-1000). Isolated RNA samples were kept at − 80 °C until use. 1 μg of total RNA samples were mixed with the PrimeScriptTM RT Master Mix (RR036A, TakaRa, China) for reverse transcription. The resulting cDNAs were diluted with distilled water at 1:5. 1 μl of the cDNA sample and the ChamQ Universal SYBR qPCR Master Mix (Q711, Vazyme Biotech, China) were used for each qPCR experiment. Q-RT-PCR was conducted using a VIIA7 Real-Time PCR System (Applied Biosystems, USA). Relative mRNA levels for *Ggps1* or *Pax6* were normalized to these for the mouse *β-actin*. Primer information was as follows. For *Ggps1*, the forward primer was TTTTGCATACACTCGACACACT and the reverse was ACCACAGGCCTCAATTTGTTTGT. For *Pax6*, the forward primer was TACC AGTGTCTACCAGCCAAT and the reverse was TGCACGAGTATGAGGAGGTCT. For *p21*, the forward primer was CCTGGTGATGTCCGACCTG and the reverse was CCATGAGCGCATCGCAATC. For *β-actin*, the forward primer was GTGACGTTG ACATCCGTAAAGA and the reverse was GCCGGACTCATCGTACTCC.

### Footprint test

Mice at P16 were used for behavioral tasks. Paws in the forelimbs and hindlimbs were coated with red and blue paints, respectively. Each mouse was allowed to walk along a restricted runway on a white paper, and its footprints were marked by red and blue colors. The stride length for the forelimbs or the hindlimbs was measured as the average distance for the red marks or the blue marks, respectively.

### Rotarod test

The method for this task was described recently [45, 46]. Briefly, each mouse was placed on a rotating rod in the Rotarod equipment (YLS-4C, Zhenghua Technology, China), facing the opposite direction of the rotation. The rotation speed was 5 rpm/min. The latency to fall from the rod was recorded for each mouse. The maximum time for each trial was 5 min. The mice were trained for 3 consecutive trials on the testing day, and the inter-trial interval was 10 min.

### Beam walking test

This task was described recently [45]. Each mouse was placed on a horizontal wooden beam, which is 70 cm in length and 30 cm above the floor. It was allowed to walk along the beam. The latency to fall from the beam was recorded for each mouse. The maximum time for each trial was set as 5 min.

### Open field test

This task has been described recently [47, 48]. Briefly, each mouse was placed in the center of a square-shaped arena measured as 30 cm × 30 cm × 45 cm. It was allowed to walk freely in the arena for 10 min. Its activities were recorded with a video camera. The time spent in the central area (15 cm × 15 cm) and the total distance traveled by each mouse were analyzed using a Zhenghua tracking system (Zhenghua Technology, China).

### Cell cultures

The cerebellum at P0 was dissected in ice-cold HBSS (Gibco) under a microscope. Tissues were then digested with an Accutase (SCR005, Millipore) at 37 °C for 20 min. After the supernatants were removed, the remaining tissues were gently triturated for 10–12 strokes. Cell suspensions were filtered using a 70 μm strainer (BD) and then separated by gradient centrifugation with Percoll solutions (30% and 65%) (GE Healthcare). Purified progenitor cells were collected from the interface and then washed with HBSS. Cells were resuspended, counted and plated in untreated 24-well plates (Corning) at the density of 500,000 cells/ml. Cells were then cultured in Neurobasal Medium (21103-049, Gibco) containing 2% B-27 (17504-044, Gibco), 2 mM GlutaMAX-I (Gibco), EGF (20 ng/ml, 236-EG-01M, R&D), bFGF (20 ng/ml, 4114-TC-01M, R&D) and 1% penicillin/ streptomycin (15140122, Gibco) in a 5% CO_2_ incubator at 37 °C. Cells were cultured for 7 days.

### Statistical analysis

Data were presented as the mean ± SEM. To analyze the expression of Ggpps across ages, one-way ANOVA (analysis of variance) was conducted using the GraphPad Prism8. On the other hand, two-tailed student’s t-test was used to examine main genotype effects between WT and *Ggps1* cKO groups. *P* < 0.05 was considered statistically significant.

## Results

### Ggpps is crucial for the development of the cerebellum

**Fig. 1:**
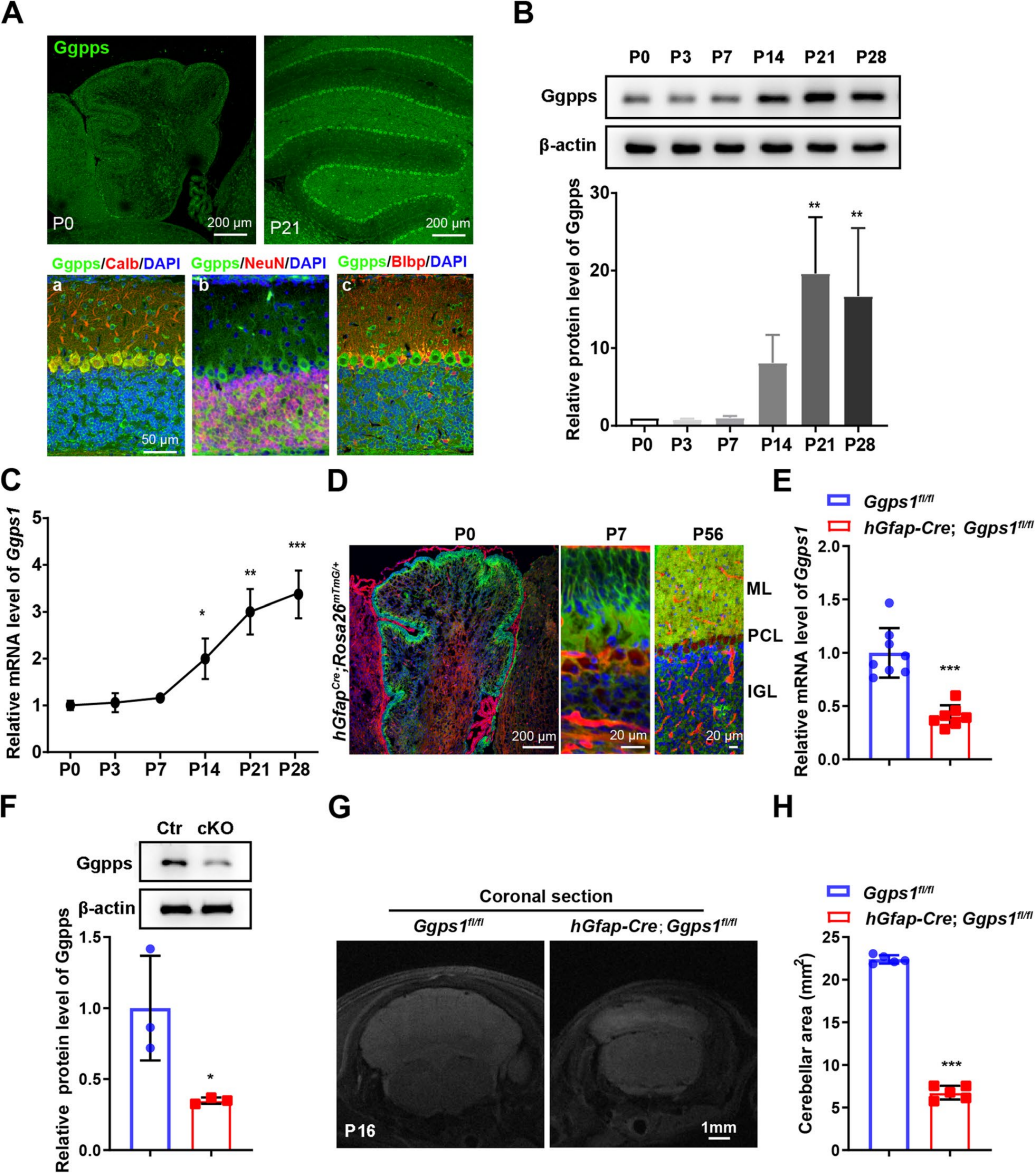
A requirement of Ggpps for cerebellar development. **A** Representative fluorescence images for Ggpps and co-staining for Ggpps/Calbindin, Ggpps/NeuN and Ggpps/Blbp in the cerebellum of a control mouse. Brain sections at P0 and P21 (a, b, c) were used. Scale bar is 50 μm in a–c. **B** Western blotting on Ggpps using cerebellar samples across ages. There was an age-dependent increase on Ggpps levels in the postnatal cerebellum (**P = 0.002, **P = 0.008, respectively; n = 3 mice per age). Raw data were shown in Additional file 2: Fig. S2A. **C** Quantitative RT-PCR analysis on *Ggps1*. There was significant increase on *Ggps1* mRNA levels in the postnatal cerebellum with aging (*P = 0.018, **P = 0.002, ***P = 0.001, respectively; n = 3 mice per age). **D** Representative images for fluorescence for GFP and tdTomato. Brain sections were prepared from *hGfap-Cre;Rosa26*^*mTmG/*+^ mice at P0, P7 and P56. Scale bar was shown in each image. **E** Quantitative RT-PCR results for *Ggps1*. There was significant difference between control and *Ggps1* cKO mice at P0 (***P = 3.0 × 10^–5^; n = 8 for WT, n = 7 for cKO). **F** Western blotting results for Ggpps. Cerebellar samples were used. There was significant difference on Ggpps between control and *Ggps1* cKO mice at P0 (*P = 0.038; n = 3 mice per group). Raw data were shown in Additional file 2: Fig. S2B. **G** Representative MRI images for control and *Ggps1* cKO mice at P16. The scale bar is 1 mm. **H** Quantification results on the area of the cerebellum from MRI images. There was highly significant difference between control and *Ggps1* cKO mice (***P = 3.1 × 10^–10^; n = 5 per mice group)

To study the role of protein prenylation in cerebellar development, we analyzed the expression pattern of Ggpps in the developing cerebellum in mice. First, we performed fluorescence immunohistochemistry (IHC) on Ggpps using brain sections prepared from control mice at postnatal day 0 (P0) and P21 (Fig. 1A). IHC results revealed abundant expression of Ggpps in the cerebellum at P0 or P21 (Fig. 1A). More specifically, we carried out co-staining of Ggpps with various markers for different types of cells, including Calbindin for PCs, NeuN for neurons and Blbp for Bergmann glia (BG)/astrocytes[10, 49, 50]. We observed strong immuno-reactivity of Ggpps in Calbindin +, NeuN + or Blbp + cells in the control cerebellum at P21 (Fig. 1A). Second, Western blotting on Ggpps was performed using cerebellar protein samples at different ages, including P0, P3, P7, P14, P21 and P28. We found that Ggpps protein levels were increased in the cerebellum of control mice with ageing (Fig. 1B). Third, *Ggps1* mRNA levels were examined using cerebellar RNA samples at the above ages with qRT-PCR. We observed increased *Ggps1* mRNA levels in the control cerebellum (Fig. 1C). Overall, the above data suggest an age-related increase in Ggpps expression in the postnatal cerebellum of control mice.

Since our recent work has demonstrated that straight knockout of the *Ggps1* gene causes embryonic lethality in mice [37, 39, 40, 51], it precludes the possibility to study the in vivo function of Ggpps in the cerebellum using *Ggps1* KOs. To overcome this problem, the human *Gfap-Cre* transgenic mouse line (*hGfap-Cre*) was used to generate viable *Ggps1* cKO mice. It has previously been shown that the expression of Cre starts as early as E13.5 in *hGfap-Cre* mice, and that Cre is expressed in NPCs in the Rhombic lip and in GCPs in the EGL in the cerebellum [10, 34, 52]. To verify this pattern, *hGfap-Cre* mice [34, 35] were crossed to *Rosa26*^*mTmG/*+^ [36, 53] to generate *hGfap-Cre; Rosa26*^*mTmG/*+^ mice. Brain sections of the latter were used for fluorescence analysis. As expected, strong green fluorescence signals were abundantly observed in cells in the EGL, ML and the IGL of the cerebellum at P0, P7 and P56 (Fig. 1D).

Since both NPCs and mature neurons may be readily detected in the postnatal cerebellum of control mice, we performed co-staining for Pax6/Ggpps, NeuN/Ggpps or Calbindin/Ggpps using brain sections at P4 and P7 (Additional file 1: Fig. S1). There was strong Ggpps immuno-reactivity in Pax6 +, NeuN + or Calbindin + cells in the control cerebellum (Additional file 1: Fig. S1). Whereas Pax6 + or NeuN + cells were negative for Ggpps in the cerebellum of *Ggps1* cKO mice, Calbindin + cells showed strong Ggpps immuno-reactivity (Additional file 1: Fig. S1). Next, we conducted qRT-PCR analysis using RNA samples from control and cKO mice at P0. We observed a significant reduction on *Ggps1* mRNA levels in the cKO group (Fig. 1E). Finally, Western blotting was conducted. There were significantly decreased levels of Ggpps in the cerebellum of *Ggps1* cKO mice compared with controls at P0 (Fig. 1F). It is likely that the residue Ggpps may be derived from PCs and a proportion of other types of cells, which do not express Cre.

To analyze morphological changes caused by deletion of Ggpps in the cerebellum, we performed MRI using mice at P16. We found that the cerebellum was largely shrunk in *Ggps1* cKO mice (Fig. 1G). Our quantification results confirmed a significant reduction in the average area of the cerebellum in the *Ggps1* cKO group compared with the control (Fig. 1H). Thus, conditional deletion of Ggpps causes defective cerebellum.

### Deletion of Ggpps leads to severe ataxia in mice

**Fig. 2:**
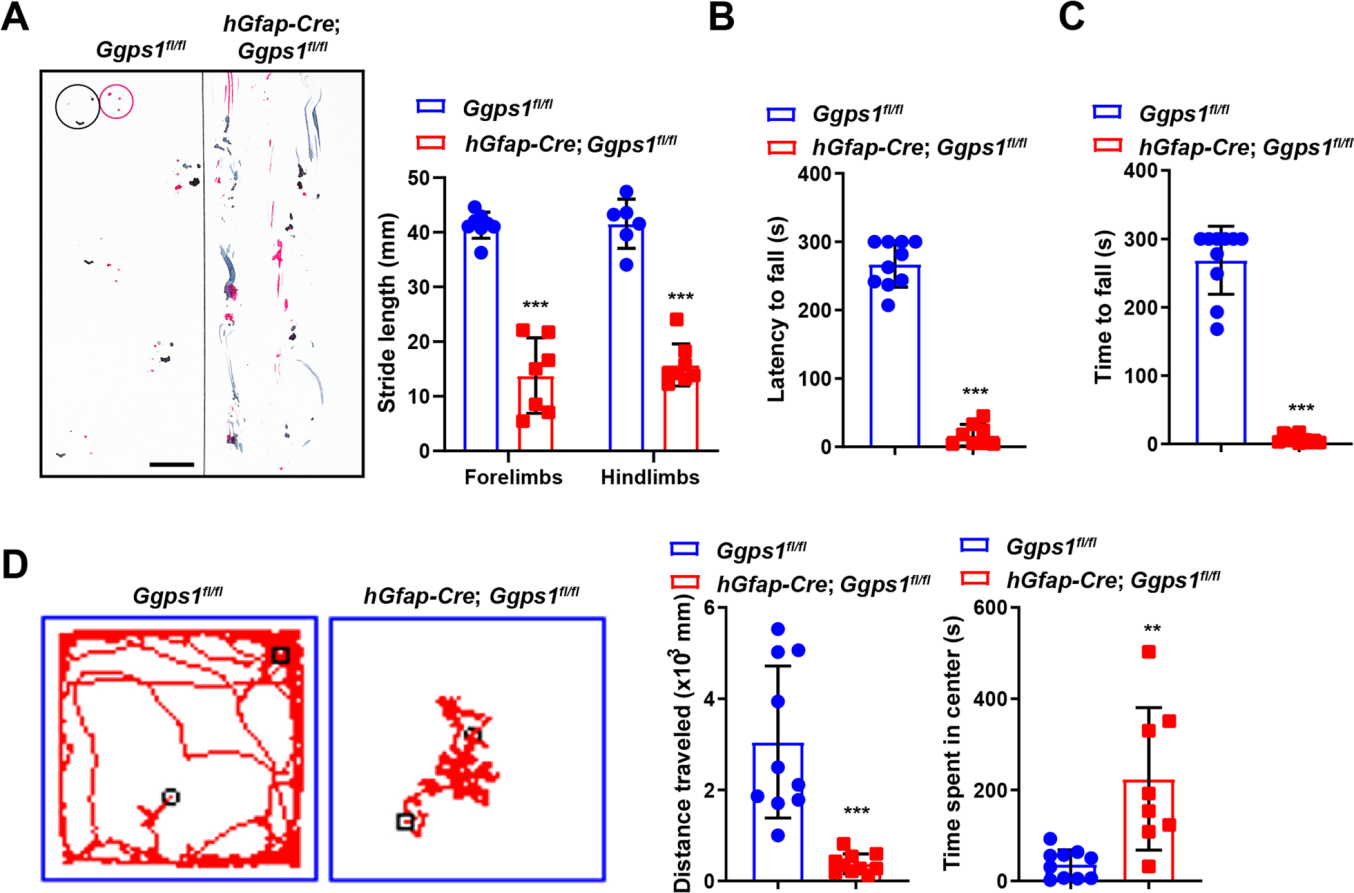
Deficient locomotor functions in *Ggps1* cKO mice. **A** Representative footprints of the mice and quantification of average footstride length. Red lines represent the paths of the fore feet, and black ones for the hind feet. Abnormal footprints were seen in *Ggps1* cKO mice at P16 and there was significant difference between control and Ggps1 cKO mice at P16 (***P = 4.7 × 10^–7^, ***P = 6.9 × 10^–8^, respectively; n = 6 for WT, n = 7 for cKO). The scale bar is 1 cm. **B** The average latency to fall in a rotarod task. There was significant difference between control and *Ggps1* cKO mice at P16 (***P = 4.1 × 10^–6^; n = 9 for WT, n = 7 for cKO). **C** The average latency to fall in a beam-walking task. There was significant difference between control and *Ggps1* cKO mice at P16 (***P = 2.5 × 10^–12^; n = 10 mice per group). **D** Performance in an open-field task. There were significant differences on the total distance traveled and the time spent in the central area between control and *Ggps1* cKO mice at P16 (***P = 9.0 × 10^–5^, **P = 0.002, respectively; n = 10 for WT, n = 8 for cKO)

We then examined whether behavior was affected in *Ggps1* cKO mice. Since we have found that *Ggps1* cKO mice started to die at about 18 days after the birth, we chose P16 as the age for behavioral testing. First, we conducted a gait test to evaluate the coordination of feet in mice at P16. We found that the pattern of the footprint from *Ggps1* cKO mice was completely disrupted compared with controls (Fig. 2A). Our results further showed that the stride length for the forelimbs or the hindlimbs of *Ggps1* cKO mice was smaller than that of controls (Fig. 2A). Therefore, the walking coordination was severely impaired in *Ggps1* mutants. Second, a rotarod test was performed. We observed that *Ggps1* cKO mice quickly fell from the rotating rod. The latency to fall from the rod was much shorter in *Ggps1* cKO mice than in controls at P16 (Fig. 2B). Thus, the motor learning was absent in *Ggps1* cKO mice. Third, a beam-walking test was carried out. In contrast to control mice, *Ggps1* cKO mice were unable to walk through the beam and fell quickly from the beam (Fig. 2C). Therefore, the balance ability was completely lost in *Ggps1* cKO mice. Finally, we conducted an open field task. Whereas control mice actively explored the whole arena, *Ggps1* cKO mice traveled a very limited distance in the arena (Fig. 2D). Quantification results showed that the total distance traveled by *Ggps1* cKO mice was remarkably lower than that by controls (Fig. 2D). In contrast, the time spent in the central area by *Ggps1* cKO mice was significantly higher than that by controls (Fig. 2D). Thus, *Ggps1* cKO mice exhibited defective locomotion in the open field.

The above results suggest that conditional deletion of Ggpps severely impairs locomotor functions in mice. However, since Cre is also expressed in radial glial progenitors, intermediate progenitors, neurons and astrocytes in the dorsal telencephalon and the hippocampus of *hGfap-Cre* mice [34, 35, 52], we reason that motor deficits seen in *Ggps1* cKO mice are likely due to all the brain sub-regions expressing Cre but not the cerebellum only. In line with this speculation, several recent studies have shown that white matter abnormality is sufficient to induce severe motor deficits in mice [43, 45, 46].

### Deletion of Ggpps causes cerebellar hypoplasia in mice

**Fig. 3:**
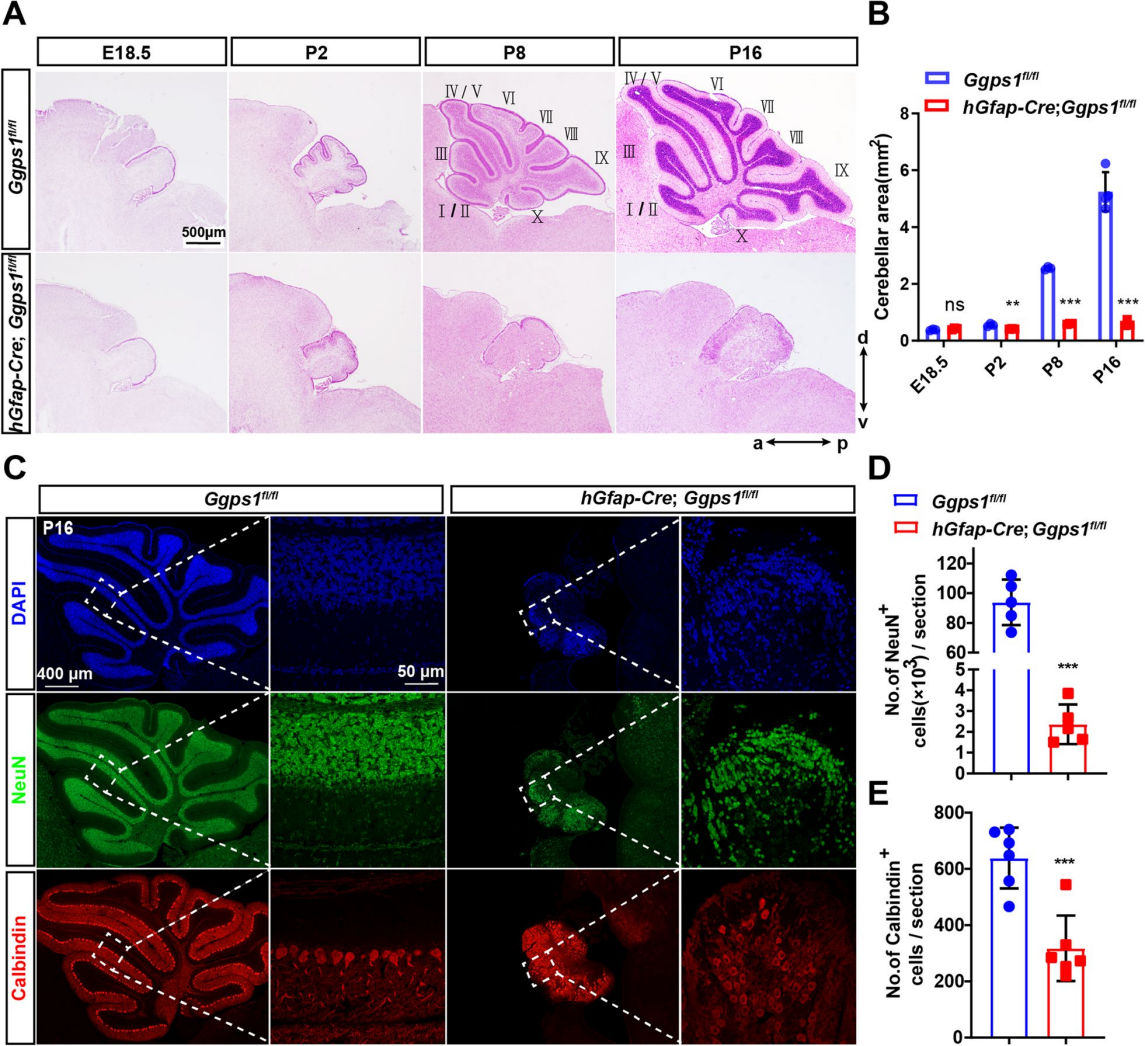
Cerebellar hypoplasia in *Ggps1* cKO mice. **A** Representative HE staining images for mice at various ages. Sagittal cerebellum sections at E18.5, P2, P8 and P16 were used. There was a severe cerebellar foliation defect in *Ggps1* cKO mice. The scale bar is 500 μm. **B** The average area of the cerebellum. There was significant difference between control and *Ggps1* cKO mice at P2, P8 or P16 (ns, no significance; **P = 0.009, ***P = 4.3 × 10^–7^, ***P = 0.001, respectively; n = 3 mice per group per age). **C** Representative fluorescence IHC images for NeuN and Calbindin. Sagittal cerebellum sections at P16 were used. The typical 10-foliation pattern was readily recognized in the NeuN + or Calbindin + sections from control mice but not *Ggps1* cKO mice at P16. The scale bars were indicated in the images. **D** The average number of NeuN + cells in the cerebellum. There was significant difference between control and *Ggps1* cKO mice at P16 (***P = 9.6 × 10^–7^; n = 5 mice per group). **E** The average number of Calbindin + cells in the cerebellum. There was significant difference between the two groups mice (***P = 0.001; n = 6 mice per group)

To investigate how loss of Ggpps affected the formation of the cerebellum, we conducted additional morphological analyses. First, HE staining was carried out using brain sections at E18.5, P2, P8 and P16 (Fig. 3A). Quantification results showed that the average area of the cerebellum was significantly decreased in *Ggps1* cKO mice across ages (Fig. 3B). Whereas the histoarchitecture of the cerebellum in the control group was highly organized, it was completely disorganized in the cKO group (Fig. 3A). In control mice at various ages, distinct lobules were readily recognized along the anteroposterior axis (Fig. 3A). Moreover, the typical 10-foliation pattern was clearly detected in control mice at P8 and P16, but it was totally missing in *Ggps1* cKO mice (Fig. 3A). There were tiny lobules in the rostral, central and caudal parts of the medial vermis in *Ggps1* cKO mice at any age indicated above (Fig. 3A). The above results suggest that Ggpps is essential for cerebellar development.

Next, we conducted fluorescence immunostaining for NeuN and Calbindin using cerebellar sections at P16 (Fig. 3C). It is known that NeuN and Calbindin are markers for GCs and PCs, respectively. First, our quantification results revealed a significant reduction on the total number of NeuN + in *Ggps1* cKO mice at P16 compared with controls (Fig. 3D), suggesting severe depletion of GCs and loss of the IGL in the cerebellum. Second, we found that the total number of Calbindin + cells was significantly decreased in *Ggps1* cKO mice compared with controls (Fig. 3E). Moreover, although control mice displayed a well-organized PCL, *Ggps1* cKO mice did not (Fig. 3C). Overall, deletion of Ggpps causes severe cerebellar hypoplasia in mice.

**Fig. 4:**
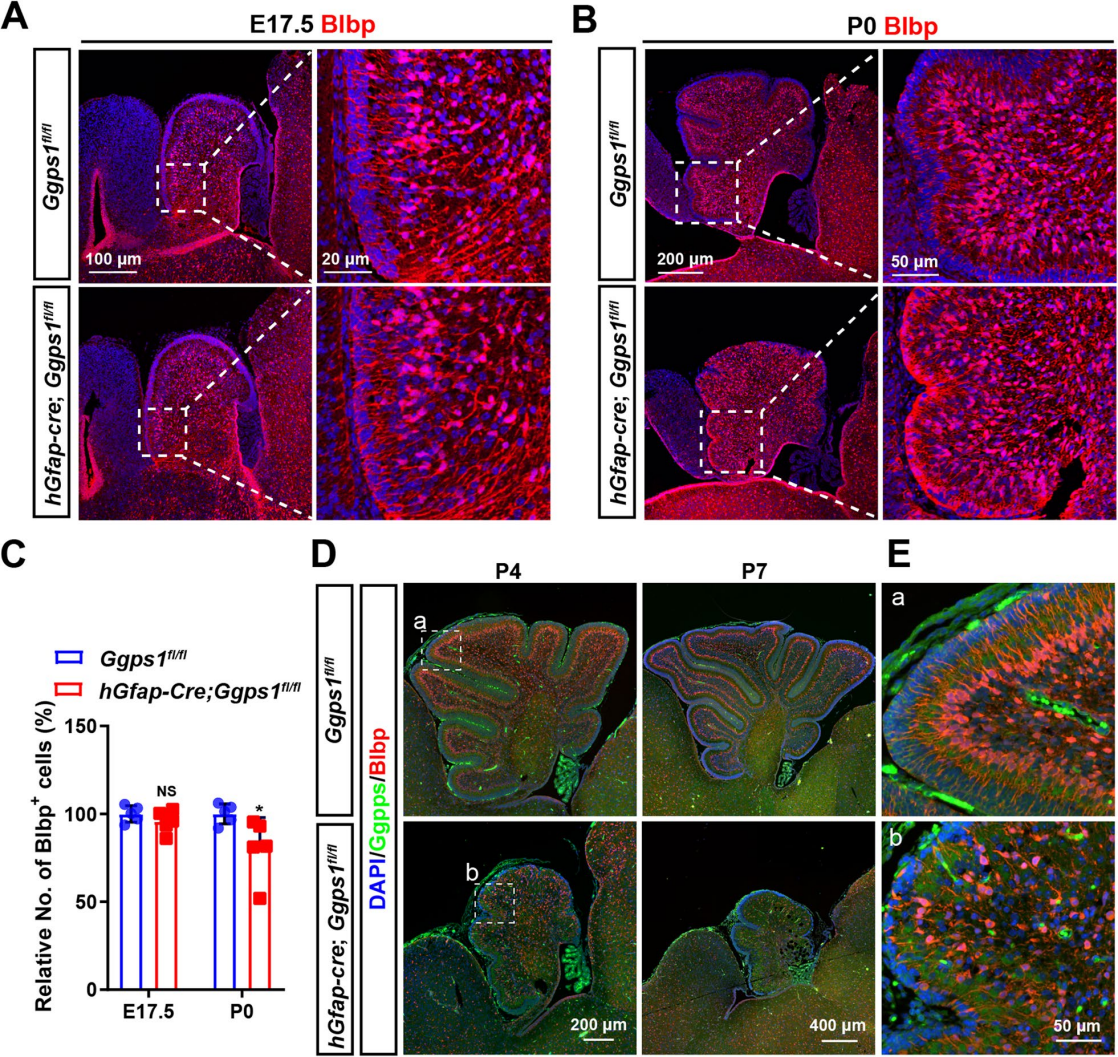
Loss of astrocytes in *Ggps1* cKO mice. **A, B** Representative fluorescence IHC images for Blbp using brain sections at E17.5 (**A**) and P0 (**B**). The scale bars were indicated in the images. **C** The average number of Blbp + cells in the cerebellum. There was significant difference between control and *Ggps1* cKO mice at P0 but not E17.5 (*P = 0.045, n = 5 mice per group at each age). **D** Representative fluorescence images for co-staining of Ggpps/Blbp on brain sections at P4 and P7. There were abundant Blbp + cells in the control cerebellum at each age. In contrast, the size of the cerebellum was very small and the population of Blbp + cells was dramatically reduced in *Ggps1* cKO mice. **E** Enlarged images for the boxed areas in **D**. The fiber of Blbp + cells (red fluorescence) was abundantly co-stained with Ggpps (green fluorescence) in the control cerebellum (**a**), but it showed almost no green signals in the cKO cerebellum (**b**)

To study whether astrocytes were affected in *Ggps1* cKO mice, we conducted fluorescence IHC on Blbp in the cerebellum, using brain sections at E17.5 and P0 (Fig. 4A, B). Cell counting results showed that the average number of Blbp + cells was significantly decreased in the cerebellum of *Ggps1* cKO mice at P0 but not E17.5 compared with controls (Fig. 4C), suggesting that Ggpps may be important for glial development. To find out whether Ggpps was inactivated in BG cells in the postnatal cerebellum of cKO mice, we performed double staining of Blbp/Ggpps using brain sections at P4 and P7 (Fig. 4D, E). First of all, the cerebellum stained by Blbp was remarkably small in *Ggps1* cKO mice compared with controls at either age (Fig. 4D). Secondly, whereas the immuno-reactivity of Ggpps was strong in Blbp + cells in control mice, it was hardly detected in Blbp + cells in *Ggps1* cKO mice (Fig. 4E).

### Deletion of Ggpps leads to the depletion of granule cell progenitors in the cerebellum

**Fig. 5:**
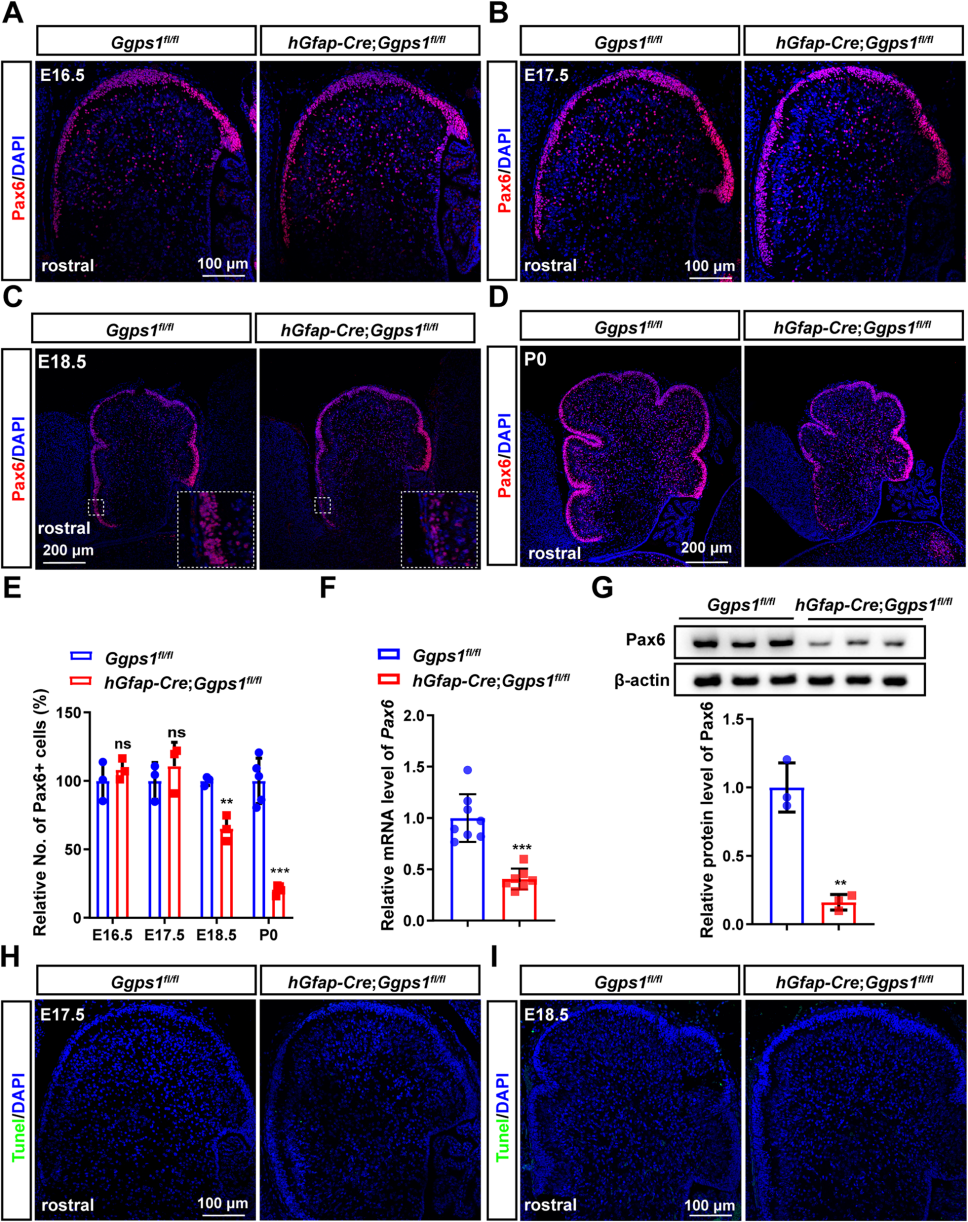
Depletion of neural progenitor cells in *Ggps1* cKO mice. **A–D** Representative fluorescence IHC images for Pax6. Pax6 labels NPCs in the EGL of the cerebellum at E16.5 (**A**), E17.5 (**B**), E18.5 (**C**) and P0 (**D**). The Pax6 + EGL was diminished in the cerebellum of *Ggps1* cKO mice at E18.5 and P0. The scale bars were indicated in the images. **E** The average number of Pax6 + cells in the EGL. There was significant difference between control and *Ggps1* cKO mice at E18.5 and P0 (**P = 0.004, ***P = 6.5 × 10^–5^, respectively; n = 3 mice per group at E16.5, E17.5, E18.5, n = 5 mice per group at P0). **F** Quantitative RT-PCR results on *Pax6*. There was significant reduction on *Pax6* mRNA levels in the cerebellum of *Ggps1* cKO mice at P0 (***P = 3.0 × 10^–5^; n = 8 for WT, n = 7 for cKO). **G** Western blotting results on Pax6. There was significant reduction on Pax6 levels in the cerebellum of *Ggps1* cKO mice (**P = 0.002; n = 3 mice per group). Raw data were shown in Additional file 3: Fig. S3A. **H, I** Representative fluorescence images for TUNEL staining using sections at E17.5 (**H**) and E18.5 (**I**). There were no TUNEL + cells in control or *Ggps1* cKO cortices. 3 embryos were examined per group per age. The scale bar is 100 μm

To find out the cellular mechanisms for the reduction of GCs observed in *Ggps1* cKO mice, we examined the population of GCPs. We used Pax6 as a marker to label GCPs in the cerebellum of *Ggps1* cKOs at different ages, including E16.5, E17.5, E18.5 and P0. Fluorescence immunostaining for Pax6 demonstrated that the immuno-reactivity of Pax6 was decreased in the EGL of *Ggps1* cKO mice at E18.5 and P0 but not E16.5 or E17.5 compared with controls (Fig. 5A–D). Cell counting results revealed that the number of Pax6 + cells in the EGL was significantly fewer in *Ggps1* cKO mice than in controls at E18.5 and P0 (Fig. 5E). Moreover, we performed biochemical analyses on Pax6. Our qRT-PCR results revealed decreased mRNA levels of *Pax6* in the cerebellum of *Ggps1* cKO mice at P0 compared with littermate controls (Fig. 5F). Western blotting data also showed a significant reduction on Pax6 in the *Ggps1* cKO cerebellum at P0 (Fig. 5G). We further performed TUNEL assay to invest whether the reduction of GCPs was due to cell death. The results showed TUNEL + cells were not observed in *Ggps1* cKO mice neither at E17.5 nor E18.5 (Fig. 5H, I). Overall, these results excluded a possibility that deletion of Ggpps may cause significant apoptotic cell death in neural progenitors in the cerebellum.

### Deletion of Ggpps impairs the proliferation of granule cell progenitors in the cerebellum

**Fig. 6:**
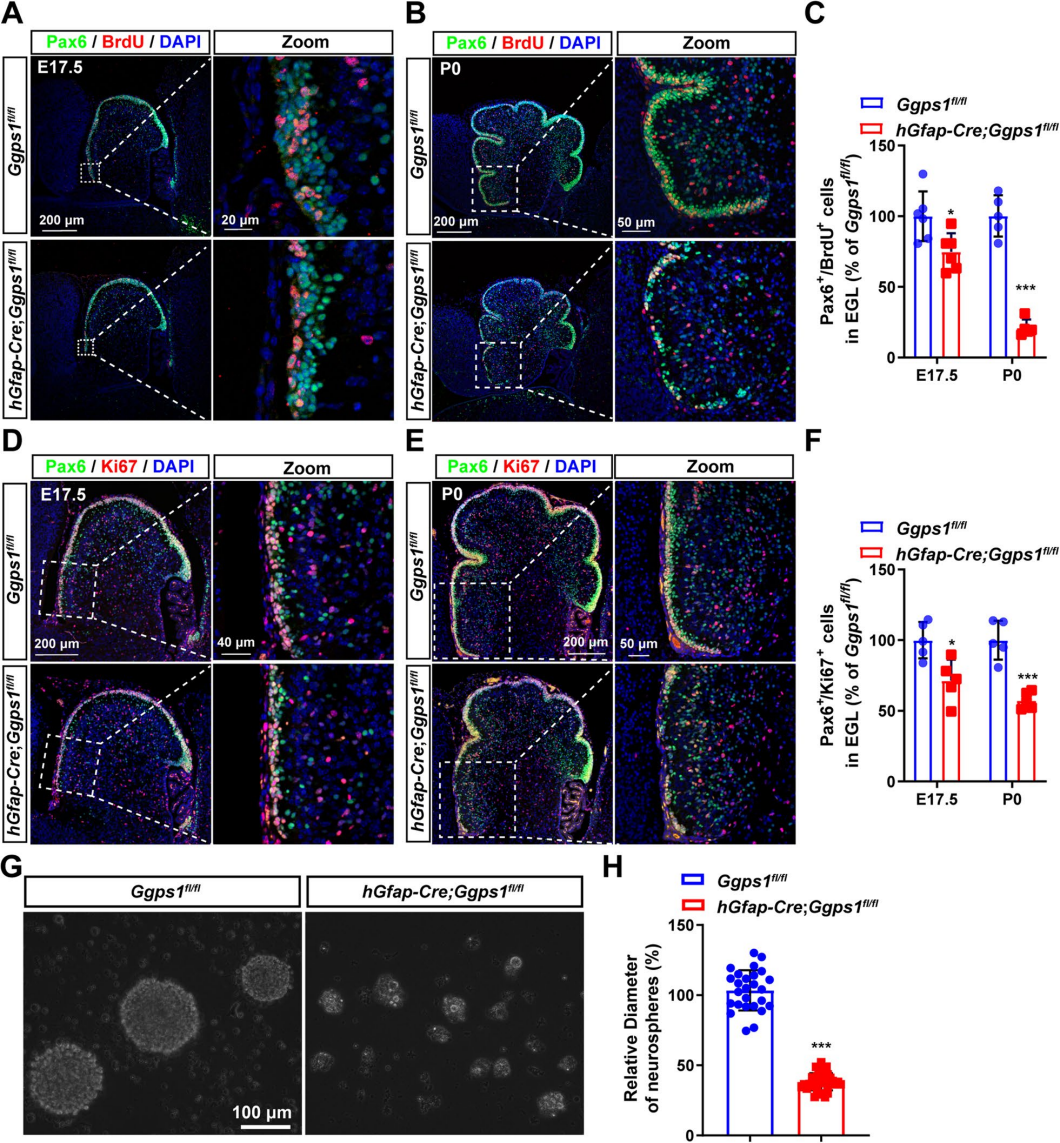
Impaired proliferation of neural progenitor cells in *Ggps1* cKO mice. **A, B** Representative fluorescence images for co-staining of Pax6/BrdU. Cerebellar sections at E17.5 (**A**) and P0 (**B**) were used. The immuno-reactivity of Pax6 + /BrdU + cells was qualitatively decreased in *Ggps1* cKO mice at P0. The scale bars were indicated in the images. **C** The average number of Pax6 + /BrdU + cells in the EGL. There was significant difference between control and *Ggps1* cKO mice at E17.5 and P0 (*P = 0.018, ***P = 3.5 × 10^–6^, respectively; n = 6 mice per group at E17.5, n = 5 mice per group at P0). **D, E** Representative fluorescence images for co-staining of Pax6/Ki67. Mice at E17.5 (**D**) and P0 (**E**) were used. The immuno-reactivity of Pax6 + /Ki67 + cells was decreased in *Ggps1* cKO mice. The scale bars were indicated in the images. **F** The average number of Pax6 + /Ki67 + cells in the EGL. There was significant difference between control and *Ggps1* cKO mice at E17.5 and P0 (*P = 0.011, ***P = 2.3 × 10^–4^, respectively; n = 5 mice per group). **G** Representative images for cultured neurospheres. Cerebellar tissues from control and *Ggps1* cKO mice were used for neurosphere cultures. The scale bar is 100 μm. **H** The average diameter of neurospheres at 7 days in vitro. There was significant reduction in the *Ggps1* cKO group compared with the control (***P = 9.9 × 10^–33^; n = 28 neurospheres cultured from 9 WT mice, n = 36 neurospheres cultured from 9 cKO mice)

To identify the mechanisms underlying the loss of GCPs in *Ggps1* cKO mice, we examined the proliferative ability of GCPs by performing BrdU pulse-labeling experiments. Since the depletion of Pax6 + cells in *Ggps1* cKOs started at the age of E18.5, we injected BrdU into pregnant mice at E17.5 and pups at P0. Brain sections were then prepared for IHC. First, double staining of Pax6/BrdU was carried out using sections at E17.5 and P0 (Fig. 6A, B). Cell counting results showed that the average number of Pax6 + /BrdU + cells was significantly decreased in the EGL of *Ggps1* cKO mice at E17.5 (Fig. 6A, C) and P0 (Fig. 6B, C) compared with controls. Second, fluorescence immunostaining of Pax6/Ki67 was performed (Fig. 6D, E). In line with findings on Pax6 + /BrdU + cells, the average number of Pax6 + /Ki67 + cells was significantly decreased in the EGL of *Ggps1* cKO mice at E17.5 (Fig. 6D, F) and P0 (Fig. 6E, F). Overall, the above results suggest that deletion of Ggpps may impair the proliferative ability of GCPs in the cerebellum.

To verify the above in vivo findings, we further performed in vitro experiments. We cultured neurospheres using cerebellar tissues from control and *Ggps1* cKO mice at P0 (Fig. 6G). We found that the average diameter for neurospheres cultured from control mice was significantly larger than that from *Ggps1* cKO mice (Fig. 6H). These in vitro results also suggest that the proliferation of cerebellar progenitors may be impaired by loss of Ggpps.

### Deletion of Ggpps reduces the geranylgeranylation of RhoA and up-regulates the expression of p21

**Fig. 7:**
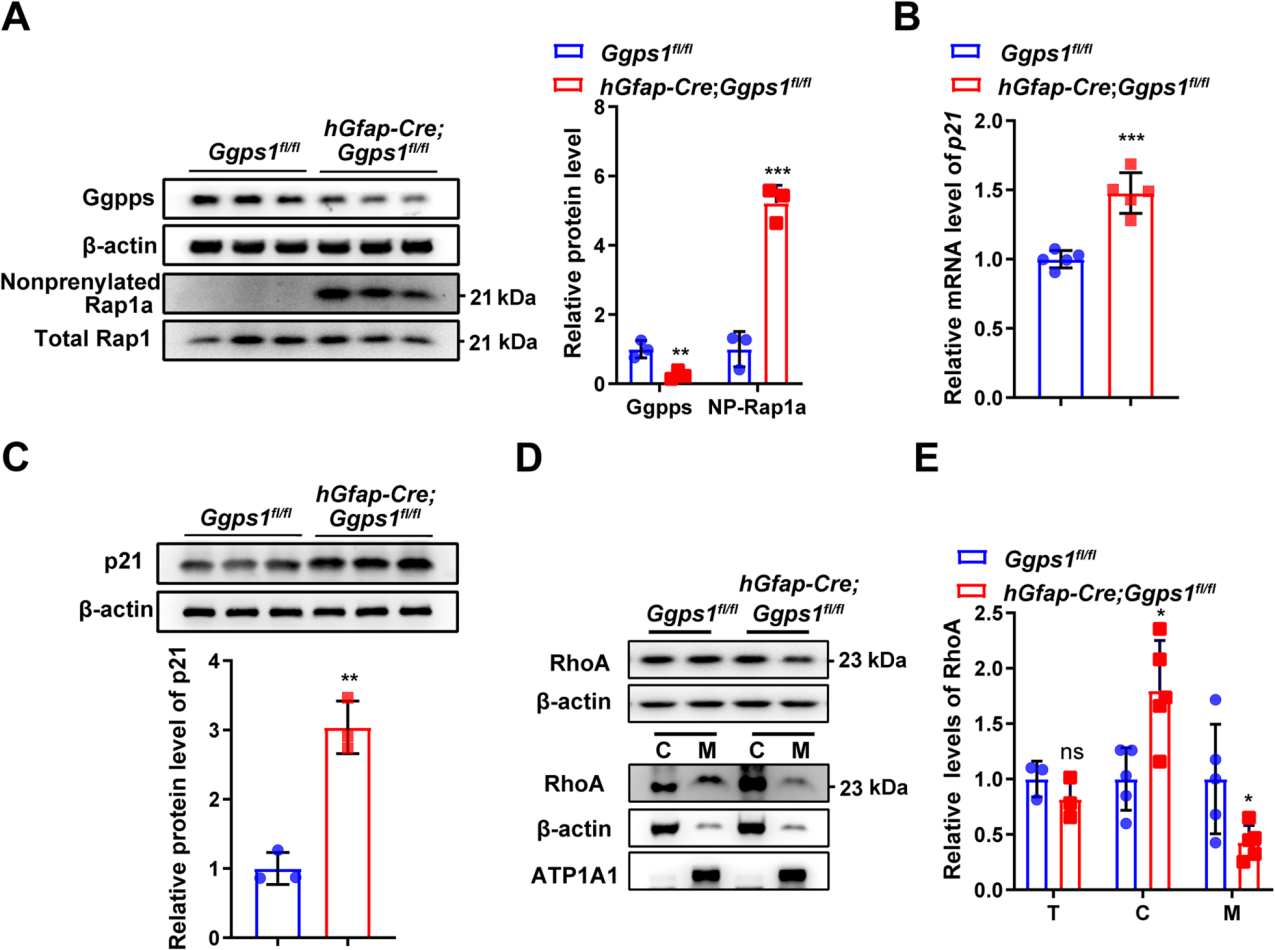
Decreased geranylgeranylation of RhoA and increased expression of p21 in *Ggps1* cKO mice. **A** Western blotting for Ggpps and non-prenylated Rap1a. There was a significant reduction on Ggpps levels in *Ggps1* cKO mice (**P = 0.010; n = 3 mice per group). There was a significant increase on protein levels of non-prenylated Rap1a in *Ggps1* cKO mice compared with controls at P0 (**P = 5.3 × 10^–4^; n = 3 mice per group). β-actin was used as the internal control. Raw data were shown in Additional file 4: Fig. S4A. **B** Quantitative RT-PCR analysis on *p21*. There was a significant increase on *p21* mRNA levels in *Ggps1* cKO mice compared with controls at P0 (***P = 1.5 × 10^–4^; n = 5 mice per group per age). **C** Western blotting results for p21. There was a significant increase in *Ggps1* cKO mice at P0 (**P = 0.001; n = 3 mice per group). Raw data were shown in Additional file 4: Fig. S4B. **D** Western blotting for Rho A. Protein lysates for total (T) cerebellum, membrane (M) and cytosol (C) fractionations were prepared from mice at P0. Raw data were shown in Additional file 4: Fig. S4C. **E** Relative levels of RhoA in different lysates at P0. There was no significant difference on levels of total RhoA between control and *Ggps1* cKO mice (*P* > 0.05, n = 5 mice per group). There were significant differences on RhoA levels in the cytosol and membrane fractionations between control and *Ggps1* cKO mice (*P = 0.010, *P = 0.039, respectively; n = 5 mice per group). β–actin and ATP1A1 were used as the internal control for the cytosol and membrane fractionations, respectively

To uncover the underlying molecular mechanisms for defective proliferation of GCPs in *Ggps1* cKO mice, the following biochemical analyses were conducted using cerebellar samples at P0. First, we performed Western blotting on Ggpps and Rap1a. The reason to choose Rap1a as a marker for protein prenylation was as follows. It is well known that Rap1a can only be modified by geranylgeranylation but not farnesylation [20]. As expected, there was a highly significant reduction on Ggpps levels in *Ggps1* cKO mice compared with controls (Fig. 7A). Our results showed that levels of non-prenylated Rap1a were significantly increased in the cerebellum of *Ggps1* cKO mice compared with controls (Fig. 7A), suggesting that deletion of Ggpps may inhibit the geranylgeranylation of proteins. Interestingly, a previous work has shown that disrupted protein geranylgeranylation may affect the G1 phase of the cell cycle [54].

It is well accepted that cyclin-dependent kinase inhibitor p21 plays an important role in the proliferation of progenitor cells [55, 56]. To study whether the expression of p21 was affected in *Ggps1* cKO mice, we first conducted qRT-PCR analysis on *p21*. We found that mRNA levels of *p21* were significantly increased in the cerebellum of *Ggps1* cKO mice at P0 (Fig. 7B). We next performed Western blotting on p21 (Fig. 7C). We observed a significant increase in p21 in *Ggps1* cKO mice compared with controls at P0 (Fig. 7C). Together, the above data suggest that p21 may be up-regulated by deletion of Ggpps.

Recent evidence has shown that RhoA may negatively regulate the expression of p21 [57, 58]. We thus examined whether or not the geranylgeranylation of RhoA was affected in *Ggps1* cKO mice. First, we performed Western blotting on total RhoA using cerebellar lysates prepared from mice at P0 (Fig. 7D). We found that the total levels of RhoA were not significantly different in the cerebellum of control and *Ggps1* cKO mice (Fig. 7E), suggesting that deletion of Ggpps may not affect the expression of RhoA. Second, membrane and cytoplasm fractionations were collected using cerebellar samples prepared from control and *Ggps1* cKO mice at P0. We then performed Western blotting using the above fractionations (Fig. 7D). Whereas levels of prenylated RhoA in the membrane were significantly decreased, those of cytosolic RhoA were increased in *Ggps1* cKO mice compared with controls (Fig. 7E). Thus, cellular localization of RhoA was significantly altered in *Ggps1* cKO mice. Overall, the above results indicated that deletion of Ggpps causes translocation of RhoA into the cytosol and may inhibit the distribution of RhoA in the membrane and other cellular compartments.

## Discussion

Protein prenylation is a process critical for various signaling pathways and human diseases [29, 37, 38]. However, it remains unknown whether protein prenylation may play an important role in the development of the cerebellum. To address this question, we generated a mutant mouse model, in which *Ggps1* is inactivated in neural progenitors and the prenylation of proteins is inhibited in the developing cerebellum. We show that deletion of Ggpps causes severe ataxia and cerebellar hypoplasia in mice. We demonstrate that deletion of Ggpps results in deficient proliferation of cerebellar GCPs. We further report that deletion of Ggpps disrupts geranylgeranylation of RhoA and leads to increased levels of p21 in the developing cerebellum.

The importance of protein prenylation in the nervous system has recently been studied. First, several studies reported that GGT1 promotes the dendritogenesis in vitro and in vivo [31, 32, 59]. Second, GGPP or geranylgeraniol (GGOH) was found to protect neurons from programmed cell death induced by statins [60, 61]. Third, GGT is plays an important role in the dendritic development of PCs [33]. Fourth, GGT and GGPP are believed to be potential targets for the treatment of neurodevelopmental disorders, including autism, depression, and schizophrenia [59]. Here, through a comprehensive analysis of the *hGfap-Cre*-mediated *Ggps1* cKO mouse, we have identified essential roles of Ggpps in the cerebellar formation and in the proliferation of GCPs. Thus, this study uncovers a novel function of protein prenylation in the cerebellum.

To dissect cellular mechanisms underlying cerebellar hypoplasia in *Ggps1* cKO mice, we examined the populations of NPCs and neurons. First, the reduction on Pax6 + cells indicates depletion of GCPs in the *Ggps1* cKO cerebellum, suggesting that Ggpps may be important for the maintenance of the GCP population. Second, we reason that massively decreased numbers of NeuN + cells may be directly due to the decrease on GCPs in the *Ggps1* cKO cerebellum. However, since BrdU birthdating experiments were not conducted in *Ggps1* cKO mice, it can not be excluded that deletion of Ggpps may also significantly affect the differentiation ability of GCPs. Third, the reduction on the numbers of Pax6 + /BrdU + cells and Pax6 + /Ki67 + cells strongly suggests that the proliferation of GCPs may be impaired by deletion of Ggpps in the cerebellum. It is quite likely that deficient proliferative capability of GCPs may directly cause a significant decrease in the population of GCPs and subsequently affect the population of neurons. We reason that the loss of GCPs and neurons may serve as the cellular mechanisms for behavioral deficits in *Ggps1* cKO mice.

To find out molecular mechanisms responsible for the change in GCPs in *Ggps1* cKO mice, we examined p21, a factor important for the cell cycle. First, we observed significantly increased expression of p21 in the *Ggps1* cKO cerebellum. Since p21 is negative regulator for the proliferation of progenitors [62], it is quite likely that increased p21 may repress the proliferation of NPCs/GCPs in *Ggps1* cKO mice. This idea is in agreement with a previous study showing that geranylgeranyltransferase 1 inhibitors induce p21 expression and inhibit the proliferation of cancer cells [63]. Since induction of p21 may be associated with G1-phase arrest [63], it is reasonable to propose that p21 may affect the proliferation of NPCs/GCPs through inducing the cell cycle arrest. Second, several studies have revealed an important role of RhoA in the proliferation of stem cells. For example, it has been shown that ablation of RhoA causes impaired proliferation of neuroepithelial cells in the spinal cord [64], and that inhibition of RhoA geranylgeranylation by simvastatin suppresses the self-renewal of embryonic stem cells [65].

In this study, we have demonstrated that Ggpps is critical for post-translational modification of RhoA. For example, we observed translocation of RhoA into the cytosol of the cerebellum in *Ggps1* cKO mice, suggesting that Ggpps-dependent post-translational modification may significantly alter sub-cellular localization of RhoA. In line with this finding, it has been shown that an inhibitor of GGPP also causes translocation of RhoA into the cytosol [65]. Moreover, previous studies have nicely demonstrated that RhoA negatively regulates the expression of p21 through a transcriptional mechanism [57, 58]. Since translocation of RhoA into the cKO cytosol can reduce levels of RhoA in the membrane, the nucleus and other cellular compartments, we speculate that the increase in p21 expression in the cKO cerebellum may be due to a RhoA-dependent transcriptional mechanism. Moreover, it has been shown that mechanisms for RhoA-dependent regulation of p21 expression are quite complex [66]. Thus, it can not be excluded that additional post-translational mechanisms may also be involved in increased p21 levels in *Ggps1* cKO mice.

Due to very limited research, physiological functions of Ggpps in the central nervous system (CNS) remain largely unknown. For the first time, this study reveals a critical role of Ggpps in the proliferation of NPCs during cerebellar development. Since our results have indicated that Ggpps is highly expressed in the postnatal cerebellum (Fig. 1), it can be expected that Ggpps may play important roles in mature GCs, PCs and other types of cells in the adult cerebellum. Moreover, it remains unknown whether Ggpps is required for mature neurons in other brain areas, e.g. the cortex, in adult mice. To address these questions, different *Cre* lines with Cre being specifically expressed in different cells need to be used to breed with floxed *Ggps1* mice to generate new types of *Ggps1* cKO mice. In contrast, since Cre is expressed in both NPCs and GCs in the cerebellum of *hGfap-Cre* mouse, the latter may not be ideal for studying roles of Ggpps in mature GCs and glial cells. Fortunately, numerous lines of *Cre* mice are widely available to researchers in the neuroscience field [1, 2, 10, 36, 41, 46, 47, 49], and they can serve as powerful tools to help uncover cell type-specific functions of Ggpps in the CNS in the future.

**Fig. 8:**
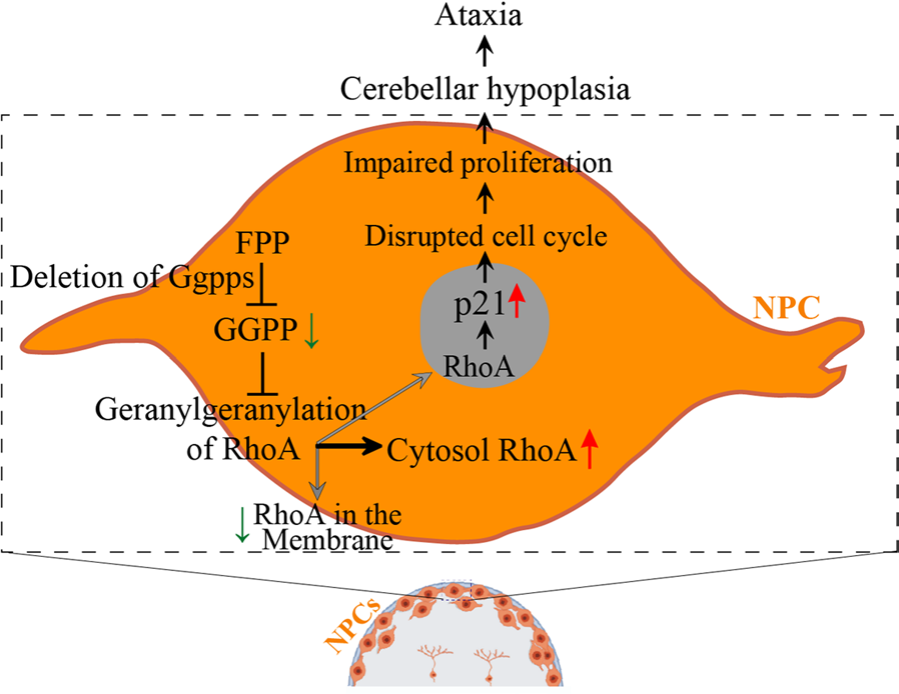
A schematic for cerebellar hypoplasia caused by deletion of Ggpps. This model depicts molecular mechanisms by which Ggpps regulates cerebellar morphogenesis. Deletion of Ggpps in cerebellar NPCs prevents the geranylgeranylation of proteins and causes abnormal prenylation of RhoA. Post-translational modification of RhoA leads to translocation of RhoA in the cytosol. Accumulation of cytosolic RhoA may reduce the distribution of RhoA in the nucleus. The latter regulates p21 expression through a transcription-related mechanism. Increased p21 then inhibits the proliferation of NPCs, which results in depletion of NPCs and GCPs. Consequently, the differentiation of neurons and glial cells is severely impaired in the cerebellum, followed by cerebellar hypoplasia and ataxia

In summary, we have identified a crucial role of Ggpps in the cerebellum in this study. To elucidate the molecular and cellular mechanisms for Ggpps-dependent cerebellar formation and function, we propose the following hypothetic model (Fig. 8). First, deletion of Ggpps causes translocation of RhoA into the cytosol via affecting the prenylation process of proteins. Second, translocated RhoA in the cytosol inhibits the distribution of RhoA in the nucleus, which then up-regulates the expression of p21, and the latter represses the proliferation of NPCs and GCPs via disruption of the cell cycle. Third, the populations of GCPs and astrocytes are subsequently decreased in the *Ggps1* cKO cerebellum. Fourth, cerebellar neurogenesis is inhibited and the population of neurons is massively decreased. Finally, cerebellar hypoplasia and severe motor deficits are displayed in *Ggps1* cKO mice.

## Conclusions

Ggpps-mediated protein prenylationin plays a critical role in cerebellar development. Loss of Ggpps impairs the proliferation of granule cell progenitors via RhoA-dependent regulation of p21. This study provides insights on cerebellar hypoplasia and ataxia caused by abnormal protein prenylation.

## Supplementary Information

**Additional file 1: Figure S1.** Expression patterns for different types of cells in hGfap-Cre-mediated knockout of Ggps1. A Representative fluorescence images for co-staining of Pax6/Ggpps in the cerebellum. Brain sections at P4 and P7 were used. Whereas there were abundant Pax6+/Ggpps+ cells in control mice, there were little Pax6+/Ggpps+ cells in Ggps1 cKO mice. B Enlarged images for the boxed areas in A. C Representative fluorescence images for co-staining of NeuN/Ggpps in the cerebellum. Whereas there were abundant NeuN+/Ggpps+ cells in control mice, there were little NeuN+/Ggpps+ cells in Ggps1 cKO mice. D Enlarged images for the boxed areas in C. E Representative fluorescence images for co-staining of Calbindin/Ggpps in the cerebellum. There were abundant Calbindin+/Ggpps+ cells in both control and Ggps1 cKO mice. F Enlarged images for the boxed areas in E.

**Additional file 2.** Unprocessed images of Western blot in Figure 1.

**Additional file 3.** Unprocessed images of Western blot in Figure 5.

**Additional file 4.** Unprocessed images of Western blot in Figure 7.

**Additional file 5.** Table of source data.

## Data Availability

The data that support the findings in this study are available from the corresponding author upon reasonable request.

## Abbreviations

Ggpps: Geranylgeranyl pyrophosphate synthase
GCPs: Granule cell progenitors
PD: Parkinson’s disease
GL: Granular layer
GCs: Granule cells
EGL: External granular layer
IGL: Internal granular layer
GGPP: Geranylgeranyl pyrophosphate
GGT: Geranylgeranyl transferase
PCs: Purkinje cells
NPCs: Neural progenitor cells
WT: Wildtype
cKO: Conditional knockout
qRT-PCR: Quantitative real-time PCR
*hGfap-Cre*: Human *Gfap-Cre*
MRI: Magnetic resonance imaging
E17.5: Embryonic day 17.5
P0: Postnatal day 0
HE: Hematoxylin and eosin
IHC: Immunohistochemistry
BG: Bergmann glia
