# Supplementary figures and images for: Disruption of protein geranylgeranylation in the cerebellum causes cerebellar hypoplasia and ataxia via blocking granule cell progenitor proliferation
Source: Mol Brain. 2023 Feb 13;16:24. doi: 10.1186/s13041-023-01010-4 (PMC9923931; doi:10.1186/s13041-023-01010-4)

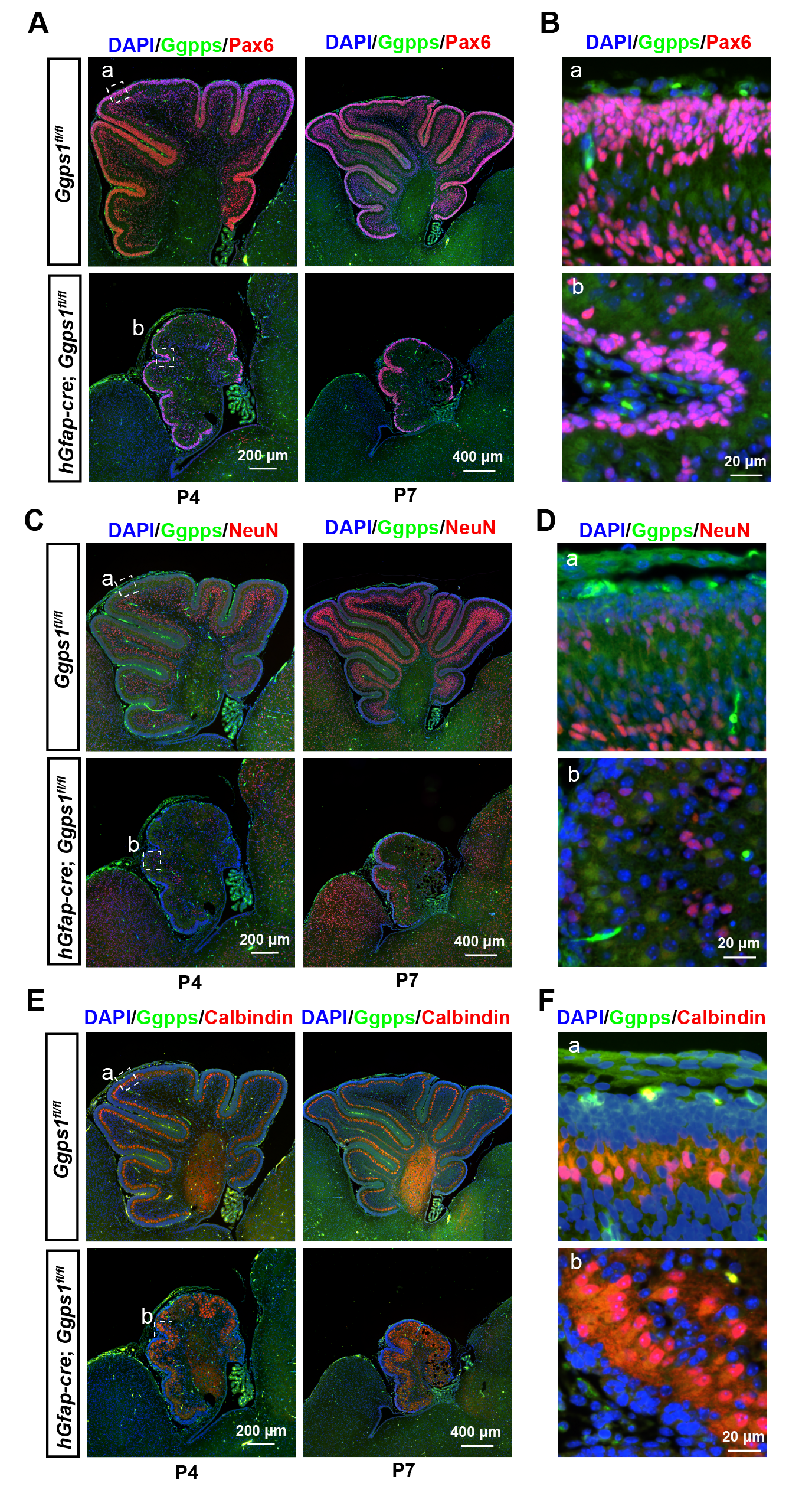

Supplement: Supplementary file 1 — Additional file 1: Figure S1. Expression patterns for different types of cells in hGfap-Cre-mediated knockout of Ggps1. A Representative fluorescence images for co-staining of Pax6/Ggpps in the cerebellum. Brain sections at P4 and P7 were used. Whereas there were abundant Pax6+/Ggpps+ cells in control mice, there were little Pax6+/Ggpps+ cells in Ggps1 cKO mice. B Enlarged images for the boxed areas in A. C Representative fluorescence images for co-staining of NeuN/Ggpps in the cerebellum. Whereas there were abundant NeuN+/Ggpps+ cells in control mice, there were little NeuN+/Ggpps+ cells in Ggps1 cKO mice. D Enlarged images for the boxed areas in C. E Representative fluorescence images for co-staining of Calbindin/Ggpps in the cerebellum. There were abundant Calbindin+/Ggpps+ cells in both control and Ggps1 cKO mice. F Enlarged images for the boxed areas in E. [file 13041_2023_1010_MOESM1_ESM.tif]

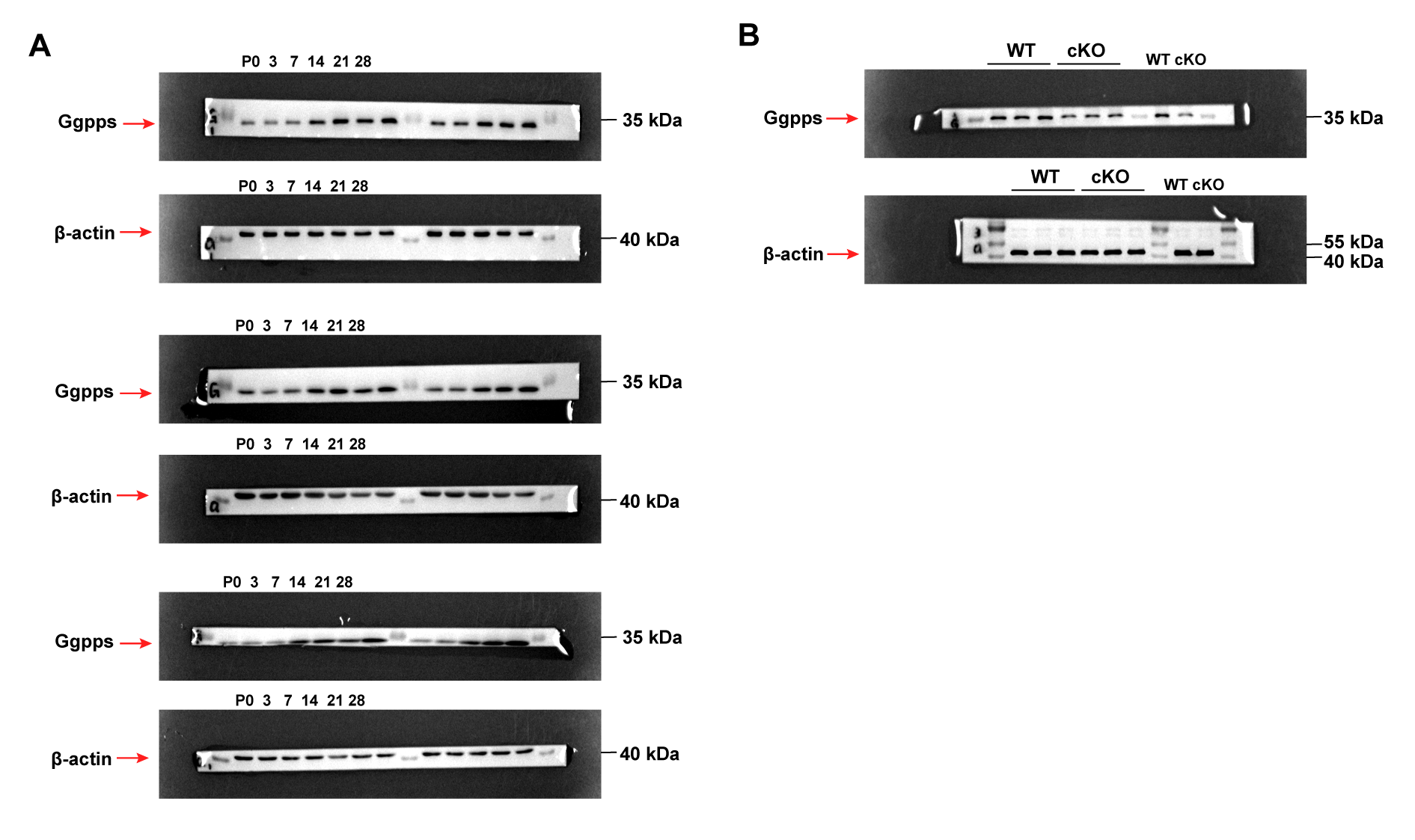

Supplement: Supplementary file 2 — Additional file 2. Unprocessed images of Western blot in Figure 1. [file 13041_2023_1010_MOESM2_ESM.tif]

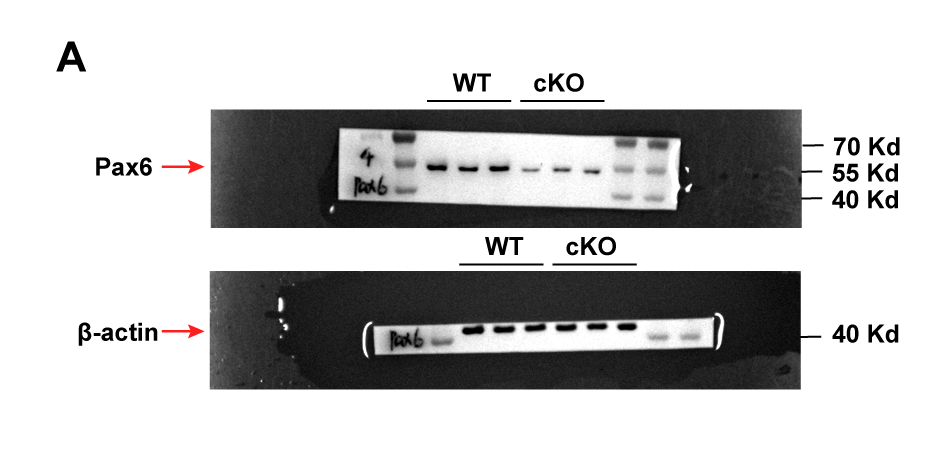

Supplement: Supplementary file 3 — Additional file 3. Unprocessed images of Western blot in Figure 5. [file 13041_2023_1010_MOESM3_ESM.tif]

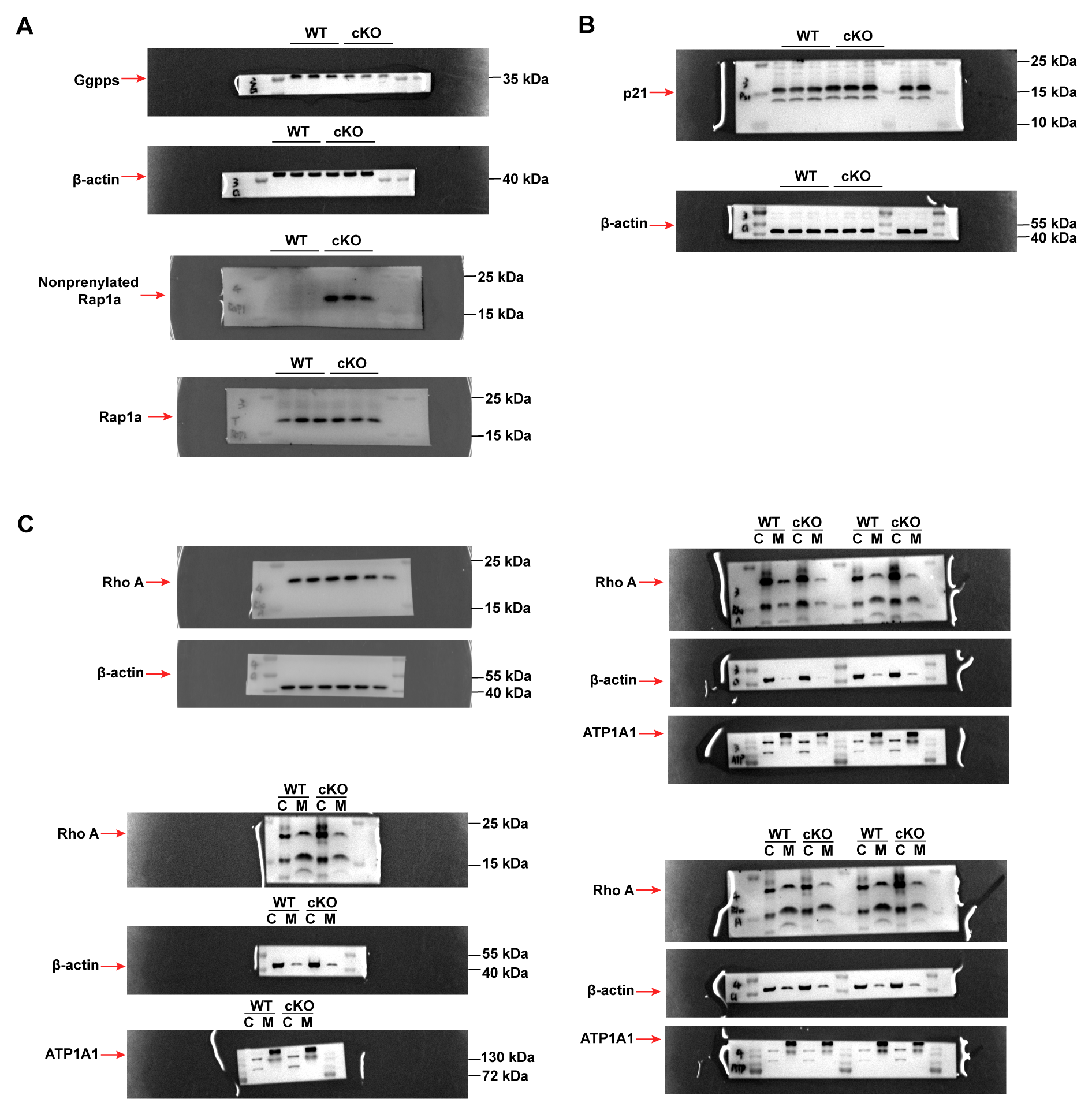

Supplement: Supplementary file 4 — Additional file 4. Unprocessed images of Western blot in Figure 7. [file 13041_2023_1010_MOESM4_ESM.tif]
